# A Retrospective Analysis of Distal Shoe Space Maintainers Placed in a Specialised Paediatric Dental Clinic

**DOI:** 10.3390/jcm15114352

**Published:** 2026-06-04

**Authors:** Rakan Hamad, Julian Schmoeckel, Christian H. Splieth, Maria Abdin

**Affiliations:** 1 Department of Paediatric Dentistry, Greifswald University, 17489 Greifswald, Germany; julian.schmoeckel@uni-greifswald.de (J.S.); splieth@uni-greifswald.de (C.H.S.); 2 Department of Oral and Maxillofacial Surgery and Plastic Operations, Greifswald University, 17489 Greifswald, Germany; maria.abdin@uni-greifswald.de

**Keywords:** distal shoe, space maintainer, premature extraction, survival rate, failure

## Abstract

**Background**: Space management after the premature extraction of second primary molars remains a challenge. Distal Shoe space maintainers (DSSMs) have been recommended after such extraction before the eruption of first permanent molars (FPMs) to prevent space loss and subsequent complications. There is lack of evidence for their clinical use and possible complications after their insertion. Therefore, this study aims to investigate survival rates and complications of DSSMs. **Methods**: Digital patients’ records were retrospectively screened for inserted DSSMs between 2014 and 2022, identifying 190 appliances in 141 children, of whom 113 appliances in 82 children had sufficient data to be included in the main outcome and survival analysis. Mean follow-up of the appliances and any reported complications were recorded. **Results**: Mean age of the included study sample (*n* = 82) at the time of insertion was 5.1 ± 0.93 years and almost identical to the whole sample (*n* = 141; 4.99 ± 0.90 years). Most children were uncooperative with high levels of caries (mean dmft: 6.6 ± 3.48). Appliances were mainly inserted under advanced behaviour management techniques such as general anaesthesia (*n* = 141, 74.2%) and nitrous-oxide sedation (*n* = 42, 22.1%). The main recorded complication was complete loss due to decementation (10.6%). Mean follow-up time was 17 months with a success rate of 83.2%. Kaplan–Meier survival analysis showed an estimated mean survival time of 42.7 months (SE 2.4, 95% CI 37.9–47.5). **Conclusions**: DSSMs were mainly placed under general anaesthesia in young children with high caries levels. The DSSM demonstrated acceptable clinical longevity till the eruption of the FPM, thus suggesting its use as a reliable option. Minor complications such as decementation could occur but are generally manageable.

## 1. Introduction

A healthy transition between primary, mixed and permanent dentition is an important part of comprehensive oral health in paediatric patients. It ensures a stable, functional permanent dentition and allows a normal dentofacial development [1]. Hence, early diagnosis and treatment of developing malocclusion is substantial for achieving a balanced and functional occlusion [2,3]. In primary dentition, caries and premature loss of primary teeth are the most common and impactful factors that often lead to notable alterations in arch integrity and alignment [4]. Despite the fact that caries is considered as a preventable and manageable chronic disease that can be diagnosed and treated in its early stages [5,6], around 50% of preschool children in different countries have experienced this disease [7]. Furthermore, caries remains the most common reason for premature extraction of primary teeth (PEPT) [8,9]. An observational study reported that second primary molars were the most commonly extracted teeth with caries being the primary reason for this intervention [10]. It has been reported that for each prematurely extracted primary tooth, an 18% increase in orthodontic treatment need in permanent dentition is to be expected [11]. In contrast, children who received continuous dental care from birth showed better oral health, including fewer prematurely lost deciduous teeth and a lower need for orthodontic treatment at the age of 8 years [12]. From that perspective, primary molars are considered to be the best space maintainers for their permanent successors [13,14] and, therefore, it is always recommended to try restoring these teeth when possible to prevent any deviations in the following dentition [15]. In cases where the extraction of the primary tooth is inevitable, managing the space and the developing dentition with a space maintainer is recommended [1,13]. The consequences of prematurely losing the second primary molars were distinctively more noticeable than the neighbouring first primary molars [16,17], and the space loss was significantly more severe in cases of premature extraction where the first permanent molar (FPM) has not yet erupted [18]. A study in Brazil recorded significant dimensional alterations in a group of children with premature loss of mandibular second primary molars after an 8-month follow-up. Authors have recommended the use of space maintainers in this situation and stated that it should be placed within the first 3 months after extraction before the maximum effect on the dental arch takes place [19]. If this situation remains unmanaged, undesirable consequences such as significant loss of arch length, midline shifts, increased risk of crowding or malocclusion that may indicate a corrective orthodontic intervention in the future may occur [4].

Therefore, the use of Distal Shoe space maintainers (DSSMs) after the early loss of second primary molars is recommended to prevent loss of space and malocclusion abnormalities in the later stages of dentition [14]. As in the cases where the loss of second primary molars occurs prior to the eruption of the FPM, the DSSM is the appliance of choice to guide its eruption [20]. Unfortunately the evidence base for use of DSSMs, and their efficacy could be described as lacking and consists of mainly expert opinions, case reports, and case series [21,22].

Since the DSSM is a temporary appliance designed to preserve arch length by guiding the eruption of the FPM following the premature loss of second primary molars, it should ideally remain functional until the FPM has successfully erupted enough that an alternative less invasive space maintainer could be inserted. Therefore, the appliance must maintain its integrity and functionality throughout this critical eruption period, usually until the age of 7–8 years. Furthermore, evaluating the performance of Distal Shoes is particularly important because these appliances are indicated at a very early age, when patient compliance is often limited compared to later stages of mixed dentition, where cooperation tends to improve and alternative space maintainers may be more suitable [23]. Consequently, understanding the longevity and failure patterns of these appliances is essential to ensure their effectiveness during this transitional phase and to support informed clinical decision-making when managing young and pre-cooperative patients.

Hence, the aim of this retrospective study is to investigate survival rates of Distal Shoe space maintainers via analysing 9 years of data regarding chairside fabricated DSSMs (Band and Loop-system) placed after the premature extraction of second primary molars in paediatric patients and to identify any reported complications that may have occurred.

## 2. Materials and Methods

### 2.1. Ethical Aspect

This retrospective study complies with the ethical standards of the institutional research committee, the principles of the Declaration of Helsinki, and the legal framework of the Landeskrankenhausgesetz Mecklenburg-Vorpommern (LKHG M-V, §§37–37d), which permits the handling of patient data for research purposes even with the absence of an individualised informed consent. On the basis of this regulation in addition to the existing approval for retrospective studies conducted at the Department of Paediatric Dentistry at the University Medicine Greifswald for assuring quality and management purposes (Internal Regulation No. BB 028/16), an additional ethical review and approval were not deemed necessary, as the study addresses a question of public relevance. Nevertheless, a written consent for the use of anonymised clinical data was routinely obtained from parents or legal guardians at the patient’s initial visit through a standardised medical history form. Only records with documented consent were included in the analysis, and all data were fully anonymised prior to evaluation.

In the course of preparing this manuscript, authors utilised ChatGPT 5 and DeepL to support improvements in grammar, spelling, phrasing, and overall clarity of the language.

### 2.2. Study Design

This retrospective study included all the inserted DSSMs after the premature extraction of second primary molars at the department of Paediatric Dentistry at the Greifswald University between 1 January 2014 and 30 September 2022. Digital patients’ records were retrospectively screened via the DAMPSOFT (© 1986–2026 DAMPSOFT GmbH, Vogelsang 1, 24351 Damp, (SH), Germany) practice management software using the service abbreviation vm066, which corresponds to the placement of fixed space maintainers. As this treatment is not covered by health insurance in Germany and must be paid for by the patient, the internal billing system was additionally cross-checked to ensure that all relevant cases were identified. Data extraction was conducted by two reviewers. The first one collected the relevant data from patients’ records using a standardised data collection protocol. Subsequently, the other reviewer examined the extracted data to verify its accuracy and completeness. Any discrepancies or conflicts between the reviewers were discussed and resolved through consensus. In cases where ambiguity remained due to incomplete or unclear clinical documentation, records were carefully reviewed jointly to reach agreement on outcome classification. Inclusion criteria were: Patients aged 2–8 years old who are healthy or are grade I or II according to the American Society of Anaesthesiologists (ASA) physical classification system [24], who have received a DSSM after the premature loss of second primary molars due to caries or undermined resorption and subsequent extraction, and the FPM has not yet erupted or just minimally erupted, where a DSSM was clinically indicated.

As DSSM is contraindicated in medically compromised patients who are grade III, IV, or V according to ASA classification, these patients would have been excluded from the study if DSSMs had been placed at all. However, no such cases were encountered during data collection. Additionally, no patients undergoing active orthodontic treatment or patients who had craniofacial developmental abnormalities were identified. Collected clinical data of patients included age at the insertion visit, gender, caries experience (decayed, missing and filled teeth and surfaces) at cavitation level (dmft/dmfs), and (idmft/idmfs) when initial caries lesions were included, extracted teeth, reported space loss, clinical setting of the extraction visit (chairside, nitrous-oxide sedation or general anaesthesia). Cooperation of the child according to the Frankl scale was also recorded, as this is considered as a standard behavioural assessment routinely performed for all patients during their initial visit to the Department of Paediatric Dentistry at the University of Greifswald.

The main outcome categories were divided as follows:

1. Successful:
  - When the DSSM was present till the full eruption of the FPM; thus, the appliance remained functional.
  - When a minor complication happened, and it was resolved by fixing the DSSM; thus, the appliance remained functional or was replaced, if the indication was given, consequently carrying out space management.
2. Failure:
  - When an event has happened in which the DSSM could neither be repaired nor replaced, resulting in its premature removal or complete loss.

### 2.3. Protocol of Placing DSSM

The Denovo Paediatric Chairside Space Maintainer System^®^ was used in this study over the entire analysed period. This system enables the treating dentist to fabricate an individualised appliance that maintains the space of primary molars in the same appointment of the extraction. The appliance consists of a band and a wire which are all made of surgical grade stainless steel. Bands are available for both maxillary and mandibular teeth in 39 different sizes and the wires are available in several varieties, including wide, narrow, plain, and Distal Shoe.

**Figure 1:**
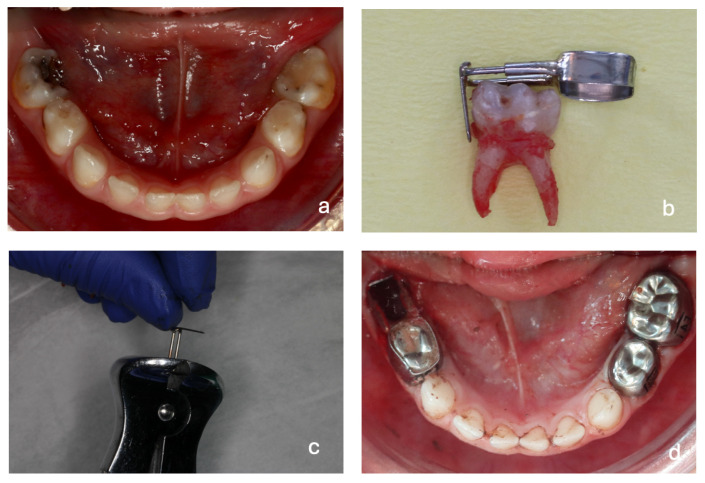
Clinical insertion of a DSSM after the extraction of mandibular right second primary molar under general anaesthesia in a 4-year-old child. (**a**) Clinical situation before treatment—tooth 85 is deeply carious with a complete destruction of the lingual wall and pulp necrosis. (**b**) After extraction, the tooth can be used to approximately measure the needed mesiodistal length of the wire. (**c**) Clipping of loop-wire with the Distal Shoe extension according to the measured distance. (**d**) Clinical situation after the cementation of the DSSM.

After evaluating the position of the second premolar, the FPM and the situation of the abutment through a pre-operative X-ray, an approximate size of prefabricated band with its attachment resting at the first primary molar adjacent to the gap is selected. Next, a wire with an appropriate width is inserted into the tubes of the band and slid or trimmed to the desired length. The wire should span the gap space and abut to the adjacent tooth. The blade of the gingival extension is then contoured with a plier to match the mesial convexity of the FPM. Finally, both tubes around the wire should be crimped with a plier and the appliance is cemented with a self-adhesive and insensitive to moisture glass ionomer cement (GC Fuji TRIAGE^®^) (Figure 1). Oral hygiene instructions are given to parents after insertion.

The patients who receive a space maintainer at the Department of Preventive and Paediatric Dentistry of Greifswald University are usually advised to report any problems that occur after the placement and get assigned check-up appointments in 3 to 6 months intervals. In follow-up appointment, when a DSSM has a complication, it is usually documented in the patient’s file and dental chart. Postoperative radiographs do not belong to the follow-up protocol at the University of Greifswald. If the DSSM remains cemented until the sufficient eruption of the FPM, it can either be altered by cutting off the Distal Shoe extension and recementing the appliance as a regular Band and Loop space maintainer or replaced completely with a new fixed or removable space maintainer.

### 2.4. Data Collection and Analysis

The search resulted in identifying 196 DSSMs placed in 144 young patients during the above-mentioned time frame. Upon screening, 4 were excluded due to being duplicates and 2 for being regular Band and Loop without distal extension but falsely registered as Distal Shoes, making the included sample for analysis of general characteristics 190 DSSMs placed in 141 children.

**Figure 2:**
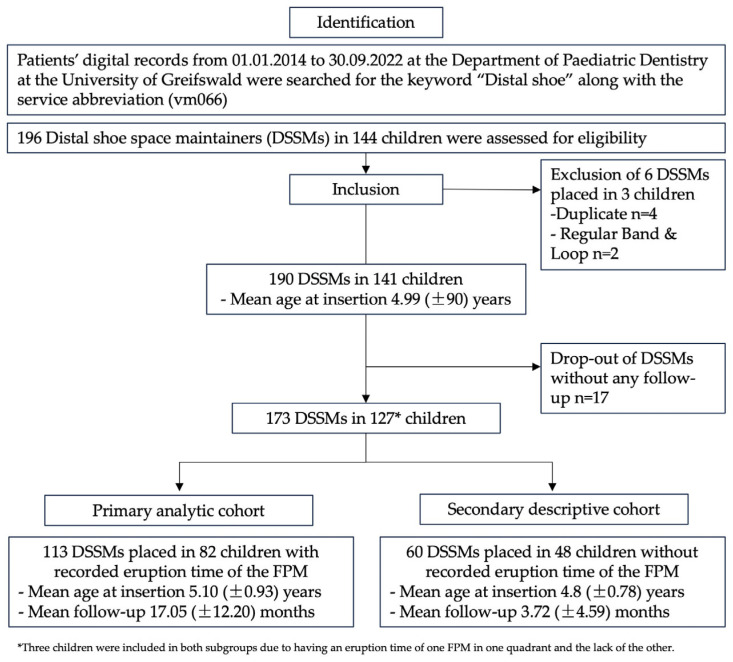
Flow chart of the study sample.

Through further assessment using the inclusion criteria, 17 DSSMs were excluded due to absolute lack of follow-up. Only DSSMs placed in children with recorded eruption time of the FPM were considered for main outcome analysis, resulting in a final sample of 113 DSSMs inserted in 82 children for analysing success and failure outcomes (Figure 2). Data were initially imported into a Microsoft Excel spreadsheet (Microsoft^®^ Excel^®^ 2013 for Windows) and then transferred to SPSS for Windows (version 30; Chicago, IL, USA) for further statistical evaluation. The independent chi-square test was used to assess statistical significance, which was set at *p* < 0.05. Kaplan–Meier survival analysis was also carried out. Descriptive statistics were reported as absolute and relative numbers.

## 3. Results

From the 144 children, who received 196 space maintainers between 1 January 2014 and 30 September 2022, 141 children with 190 space maintainers were included for the general characteristics analysis. Seventeen DSSMs were then excluded due to lack of follow-up records.

For further analyses at the space maintainer level, the remaining 173 DSSMs were then divided into two subgroups depending on whether the eruption time of the FPM was recorded or not. For outcome analysis, the subgroup containing 113 DSSMs placed in 82 children with recorded eruption time of FPMs was identified as the primary analytic cohort (Figure 2).

### 3.1. General Characteristics of the Study Sample

The number of females in the study sample was slightly higher (53.9%) than the number of males. The majority were healthy children aged between 2 and 7 years old. Patients’ mean age when the Distal Shoe was inserted was 4.99 (±0.90) years (Table 1). The majority of children (*n* = 61, 43.2%) showed low levels of cooperation according to Frankl behaviour rating scale. Mean dmft/dmfs (decayed, missing and filled primary teeth/surfaces) was 6.6/14.13 (±3.48/11.26) and when initial lesions were included, the mean idmft/idmfs increased to 8.25/16.05 (±3.97/11.58; Table 1).

Most of the DSSMs were placed under general anaesthesia (*n* = 141, 74.2%) followed by nitrous-oxide sedation (*n* = 42, 22.1%) and only very few chairside. Interestingly, space maintainers were placed more frequently in the 3rd quadrant followed by 4th, 2nd and 1st respectively. Most appliances were placed on first primary molars treated with a stainless steel crown (SSC; 58.4%) either using the (modified) Hall technique (32.6%) [25] or in the combination pulpotomy or pulpectomy with a conventional SSC (25.8%). Other appliances were cemented fully or partially on sound tooth structure (41.6%; Table 2).

#### 3.1.1. Study Sample with No Recorded Eruption Time of FPMs

In 48 children (27 males and 21 females) who received 60 DSSMs, the eruption time of the FPM was not recorded. Most of these children were uncooperative (*n* = 22, 45.8%). The mean age at insertion in this group was 4.8 (±0.78) years with a mean dmft of 7.29 (±3.28) and idmft of 8.9 (±3.56). The majority of DSSMs were inserted at the age of 5 years (40.0%). Fifty-two DSSMs (85.7%) were inserted under general anaesthesia. The overall mean follow-up was 3.72 (±4.59) months. Forty-eight of these DSSMs had a mean follow-up of 3.39 (±4.65) months and no recorded complications. The remaining 12 were lost mainly due to decementation and had a slightly longer mean follow-up time of 5.03 (±4.2) months.

#### 3.1.2. Study Sample with Recorded Eruption Time of FPMs

This group consisted of 113 DSSMs placed in 82 children (34 males and 48 females). The mean age at insertion was 5.10 (±0.93) years. Most of these children were uncooperative (*n* = 34, 41.4%). The majority of DSSMs were inserted at the age of 5 (54.0%) years followed by the age of 4 (19.5%), 6 (15.9%), 7 (7.1%), 3 (2.7%) and 8 (0.9%), respectively. The mean dmft and idmft were 6.72 (±3.58) and 8.56 (±4.15) respectively. No statistically significant difference was found when patient-level characteristics were compared between both study samples with and without eruption time of the FPM (*p* > 0.05).

Eighty-one DSSMs (71.7%) were inserted under general anaesthesia, 28 (24.8%) under nitrous-oxide sedation and four chairside (3.5%). The mean age at eruption of the FPM was 6.27 (±0.87) years. The mean follow-up time was 17.0 (±12.2) months. An orthodontic consultation was carried out in 18 (22%) children due to reported space loss.

### 3.2. Treatment Evaluation

In total, 94 (83.2%) DSSMs were successful. Minor complications occurred in 36 (31.9%) DSSMs but were either repaired or replaced; thus, the space maintenance was carried on. Failure was reported in 19 appliances (16.8%). Most common reason of failure was the complete loss of the space maintainer due to decementation followed by loss of the abutment tooth (Table 3).

The reported minor complications in 36 (31.9%) DSSMs identified in this study were:

- Reduced stability of 14 DSSMs due to partial loss of cement, which was mended by application of cement on the occlusal surface without having to remove the appliance.
- Gingival inflammation was reported in three DSSMs as pain, swelling, redness, and bleeding, which was treated with proper cleaning and parental instructions of oral hygiene.
- Nineteen DSSMs were altered to serve as fixed space maintainers consisting of Band with regular loop or replaced with a new fixed or removable one, as the FPM had already erupted and space management can be further carried on using other types of space maintainers.

The reported reasons for failure in 19 (16.8%) DSSMs with no replacement identified in this study were:

- Complete dislodgement and loss of the DSSM (10.6%).
- Premature loss of the tooth acting as abutment of the DSSM (3.5%).
- Poor fit and breakage that could not be repaired with alterations to the body of the Distal Shoe (2.7%).

### 3.3. Survival Analysis

**Figure 3:**
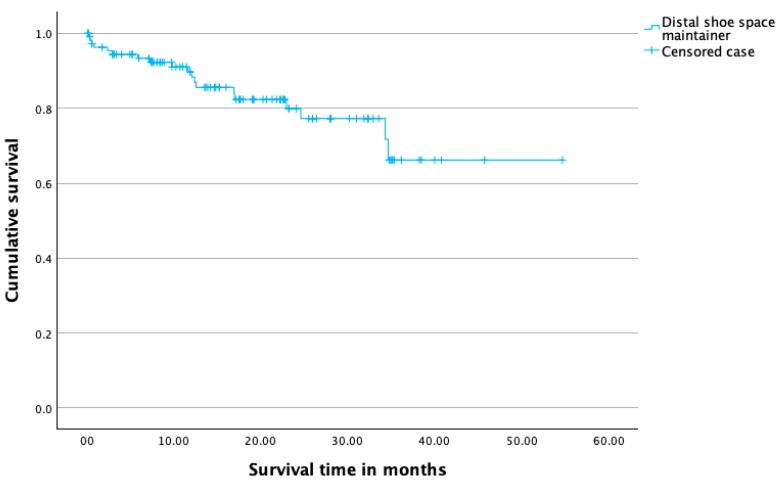
Kaplan–Meier survival plot of Distal Shoe space maintainers in a specialised care setting (*n* = 113).

For the Kaplan–Meier survival analysis of the DSSMs with reported eruption time of the FPM, the event of interest was defined as the occurrence of failure and censored cases are the successful DSSMs, which did not experience the event of failure for the duration of the study. At the 24 months marker, survival probability drops just below 80% with 30/113 cases remaining at risk (Figure 3, Table 4). Overall, the estimated survival time was 42.7 (SE 2.4, 95% CI 37.9–47.5) months.

## 4. Discussion

To the best of our knowledge, the existing literature on DSSMs consists primarily of case reports and case series with a low quality of evidence, focusing on clinical description rather than providing evidence-based data on the effectiveness or long-term outcomes of Distal Shoe appliances. Although a recent systematic review described the DSSM as an effective tool in guiding the eruption of FPMs with 95.5% successful eruption of FPMs and relatively low complication rates, the evidence base consists mainly of one retrospective cohort study with 38 patients and case series and case reports including only 29 patients in total. Hence, a stronger conclusion is drawn than what the available evidence can reliably support [26]. The present study analysed almost double the amount of DSSMs with a follow-up duration almost as twice as long. While the primary conclusion of the systematic review centres around the clinical effectiveness of DSSMs in guiding the eruption of FPMs, our study instead emphasises the clinical stability and survival rate during the critical period in which the DSSM must remain in situ to achieve the desired purpose. The present study also offers an exact report of the insertion procedure, which retrospectively addresses the concluded need mentioned in the systematic review for studies with larger cohorts to determine the best procedural parameters.

Although the survival of regular Band and Loop space maintainers has been more intensely studied in the literature [27,28,29], no previous studies have evaluated the performance of DSSMs independently from other space maintainers even though there are clear differences between the clinical indications and patient profiles such as the age of the patient [30].

### 4.1. General Characteristics of the Study Sample

The majority of the DSSMs were placed in children that were treated under general anaesthesia and nitrous-oxide sedation (*n* = 183, 96.3%). This could be explained by the young age of the patients, their low cooperation level and the extensive treatment need (Table 1). Although general anaesthesia should not be considered as the first treatment choice in children, the need to extract multiple teeth and the limited cooperation are valid indications for its use [31].

The caries experience of the studied group showed about a four-fold higher dmft when compared to the German 3- and 6–7-year-old children [32], which is representative of the patient profile typically seen in specialised paediatric dentistry settings [33]. Such a patient’s profile is likely to be generalisable to any paediatric specialist practice that offers treatment under general anaesthesia for uncooperative children with Early Childhood Caries (ECC), thus highlighting the need to offer DSSMs as the standard technique for space management in primary dentition [34,35]. However, this patient’s profile may not accurately capture other paediatric populations in other healthcare system settings with different structure and different approaches to the usage of general anaesthesia.

### 4.2. Follow-Up

According to the literature, the average eruption age of FPMs is 6.1 years in girls and 6.3 years in boys, which is in coherence with the presented results in this study [36]. Therefore, the mean follow-up duration of 17 months, in which the DSSMs were functional, can be considered as appropriate given that the mean age of the children with recorded eruption time of FPMs in this study was 5.10 (±0.93) years, which is also in line with another study that evaluated the efficacy of Distal Shoes [26].

Only nineteen (16.8%) of the included DSSMs experienced failure and were not replaced or altered (Table 3). Iatrogenic errors during insertion such as bad adaptation of band, wrong choice of size or insufficient moisture control could have likely contributed to the complete loss of the appliance. Another explanation could possibly be the limited acceptance of the appliance as a foreign object in the oral cavity, which encourages the children to repeatedly tamper with the space maintainer, leading to its distortion, especially when bearing in mind how invasive the extraction and the placement of the DSSM are.

The poor cooperation levels observed in the studied sample, along with the reported higher dental fear and anxiety levels among children who experienced general anaesthesia [37], may also have prevented the dentist during the follow-up visits from identifying and repairing what could have been minor issues, allowing them to progress into failure, and might also have made the replacement of the DSSM challenging.

Children with no recorded eruption time of the FPMs had a substantially shorter mean follow-up period of approximately 3 months. This may be explained by the referrals from other dentists in the surrounding cities for specialised dental treatment with advanced behaviour management. Subsequent follow-up visits may have taken place at another dental practice, resulting in the short follow-up period and the unclear outcome. Hence, separate analysis of this subgroup was performed to minimise any potential bias in the survival estimates of the DSSMs. Although there were no differences between the two groups regarding age, cooperation and the severity of the disease (dmft/idmft), there is still a possible selection bias to be acknowledged as the children of this subgroup might have a different socioeconomic status or a bigger travel burden, which did not allow them to attend follow-up appointments. Several studies have addressed the issue that the distance to the clinics plays a part in not attending recall visits [38,39].

### 4.3. Treatment Evaluation

The studied sample was divided into two subgroups and analysed according to the presence or absence of the recorded eruption time of the FPMs. The rationale behind this division, apart from the above-mentioned reason, is that the outcome of the DSSM is dependent on the presence of the FPM and it allows a more reliable assessment of the longevity of the appliance as the main function of the Distal Shoe is to prevent the mesial migration of the FPM.

The main recorded reason for failure was complete loss due to decementation of the space maintainer (Table 3). According to a previous study, this finding might be attributed to the weak mechanical bond between the glass ionomer cement and the SSC in the cases where the band was cemented on an SSC, which was found to be much weaker than the chemical bond of the cement with the natural surface of the tooth [40]. The reported decementation as main reason for failure is consistent with findings from numerous studies in the literature which evaluated the longevity of different types of space maintainers [27,41]. Furthermore, a considerable lever effect at the most distal point of the DSSM could be anticipated, especially if the upper antagonist is present.

Gingival inflammation was recorded only in three cases in this study. This could be due to poor band/wire adaptation and/or bad oral hygiene and thus accumulation of plaque. The recorded failure rate of 16.8% (*n* = 19/113) reflects the difficulties of maintaining DSSMs and emphasises the need for shorter recall intervals alongside the regular recall due to high caries risk, for an opportune modification or replacement of appliances, and careful selection of the most suitable subsequent space maintainer design as the FPM erupts.

It is important to keep in mind that space maintenance after the premature extraction of the second primary molar is a sequential process. It begins in the primary dentition and lasts until the permanent successor erupts at a late stage of mixed dentition phase II. DSSMs represent the first step in this process when second primary molars are extracted before the eruption of the FPMs. In the present study, DSSMs that required alteration or replacement following complications were still considered successful, as such modification would normally occur during routine follow-up once the FPM erupts, which is the clinical indication for transitioning to another type of space maintainer. On the other hand, appliances that have had complications such as loss or breakage but were not replaced or modified were classified as failures. The inability to alter or replace these appliances may have been related to persistent poor patient cooperation or to a not yet erupted FPM. From a clinical perspective, such failures represent more than temporary technical complications, as they may increase the risk of space loss due to mesial migration and tipping of the erupting FPM.

Clinical success in space management ideally refers to the effective prevention of space loss, which requires longitudinal studies that follow patients through the different stages of dentition. However, for this endpoint to be achieved, a proper adaptation, placement and stability of the DSSM must be granted. A lost Distal Shoe before the eruption of the FPM cannot be re-inserted and mesial drifting is most likely to occur. Given the retrospective design of the study, space analysis was not possible. Nevertheless, the DSSM demonstrated suitable clinical longevity, which reflects its stability and suitability as a space maintaining device before the eruption of first permanent molars. Thus, the term of success in the present study has been used to describe the DSSMs that absolutely survived or were replaced/altered after a minor complication when the FPM had already erupted, although the latter could also be interpreted as a normal clinical transition rather than successful complication management.

Although space loss was reported in 18 patients (22%), it cannot be determined whether it was caused by failure of the Distal Shoes, occurred despite their presence, or was related to or worsened by pre-existing malocclusion.

Some of the documented complications may be contributed to the lack of cooperation level of the children. These findings could, therefore, support the statement from Hicks that poor cooperation represents a contraindication for Distal Shoes [42]. However, considering a removable appliance as an alternative may also be unreliable, as previous studies reported suboptimal compliance in wearing removable space maintainers or orthodontic appliances often due to pain and discomfort [43,44].

Another possible treatment approach would be delaying the space management until the eruption of the FPM and regaining the lost space later. This strategy is particularly suitable in medically compromised patients, where the Distal Shoe is contraindicated [4]. Nevertheless, this orthodontic procedure is complex, time-consuming and costly.

### 4.4. Survival Analysis

The survival rate recorded in this study was similar when compared to that of other types of fixed space maintainers, such as Band and Loop. A previous study also showed 80% survival rate at the 20 months follow-up marker. However, the mean age of children in that study was slightly higher (5.90 years), which makes the survival outcomes observed in the present study at least as favourable [27]. It should be highlighted that the observed estimate survival time of 42.7 months is model-dependent and exceeds the actual observation period for most participants (mean follow-up time 17.0 (±12.2) months); therefore, corresponding data should be interpreted cautiously (Table 4). Moreover, the reported survival rate in this study is also restricted to the above-mentioned patients’ profile and cannot be applied to more cooperative children treated routinely chairside.

### 4.5. Limitations and Recommendations

The findings of this study must be considered within the context of its retrospective design. Dependence on existing records may have resulted in incomplete or inconsistent data. Due to the design of the study, it was not possible to collect any accurate data on the recorded space loss and actual clinical effectiveness of the DSSM in preventing or reducing the need for future orthodontic treatment.

Another important consideration is the clustering of appliances within the same child. Although this reflects real-world setting, analysis based on appliance level may however violate independence assumptions.

Given that decementation of the appliance remains the most common cause of appliance loss, there is a need for well-designed split-mouth studies to investigate if luting agents play a role in the clinical longevity of these appliances and to identify the most suitable luting agent. Possibly, other cements or materials should be considered for cases when Band and Loops with Distal Shoe extension are placed on SSCs.

Nonetheless, e.g., using a stainless steel crown and a loop with a Distal Shoe extension as a prefabricated Crown and Loop system instead of a band, as used in this study, has the advantage of better retention and very low risk of decementation. However, there is a higher risk of breakage [45] and when the FPM erupts, removal of the Distal Shoe extension is difficult, which is important to consider in such uncooperative patients.

Furthermore, future clinical trials should investigate space changes with and without Distal Shoe appliances, the position of the FPMs before and after eruption and any future need for orthodontic treatment to truly determine if the hassle of this treatment option in such specific patients’ profiles is worthwhile. In addition to clinical effectiveness, patient comfort and acceptance should also be investigated with appropriate validated questionnaires or visual analogue scales, while periodontal response can be objectively assessed using established gingival and plaque indices around abutment teeth.

## 5. Conclusions

As demonstrated in this study, Distal Shoe space maintainers are primarily placed in preschool children with high caries experience and low levels of compliance, mostly under general anaesthesia. The findings indicate that a mean follow-up of 17 months is appropriate, allowing sufficient time for the eruption of the first permanent molar, after which the Distal Shoe may be modified or replaced. The anticipated 80% survival rate in this period encourages the use of prefabricated DSSMs as the initial phase of space management when early intervention in the primary dentition is required.

## Figures and Tables

**Table 1:** Patient-level characteristics of the entire study sample (*n* = 141).

Category	Factor	Total
Gender	Female	76 (53.9%)
	Male	65 (46.1%)
Health condition	Healthy	138 (97.9%)
	Chronically ill	3 (2.1%)
Age group (in years)	2–3	16 (11.3%)
	4–5	111 (78.8%)
	6–7	13 (9.2%)
	>7	1 (0.7%)
Child cooperation at first visit Frankl scale	Uncooperative	61 (43.2%)
	Slightly uncooperative	31 (22%)
	Somewhat cooperative	40 (28.4%)
	Cooperative	9 (6.4%)
Mean age in years (±SD)		4.99 (±90)
Mean baseline dmft (±SD)		6.6 (±3.48)
Mean baseline idmft (±SD)		8.25 (±3.97)
Mean baseline dmfs (±SD)		14.13 (±11.26)
Mean baseline idmfs (±SD)		16.05 (±11.58)

SD: Standard Deviation. dmft: decayed missing filled teeth.

**Table 2:** Treatment characteristics for insertion of Distal Shoes in the study sample (*n* = 190).

Category	Factor	DSSM *n* = 190 (100%)
Clinical setting of the placement	Chairside	7 (3.7%)
	Nitrous-oxide sedation	42 (22.1%)
	General anaesthesia	141 (74.2%)
Jaw and quadrant	Upper	63 (33.1%)
	right	28 (14.7%)
	left	35 (18.4%)
	Lower	127 (66.8%)
	right	55 (28.9%)
	left	72 (37.9%)
Abutments situation	Hall-Crown	62 (32.6%)
	SSC with pulpotomy	48 (25.3%)
	No treatment	44 (23.2%)
	Filling	22 (11.6%)
	Non-cavitated arrested caries	13 (6.8%)
	Pulpectomy	1 (0.5%)

**Table 3:** Complications and failure reasons of DSSMs with reported eruption time of FPM.

Category	Factor	DSSMs *n* = 113 (100%)
Success	Absolute	58 (51.3%)
	Resolved after minor complication	36 (31.9%)
	Total	94 (83.2%)
Failure	Complete loss due to decementation	12 (10.6%)
	Premature loss of abutment	4 (3.5%)
	Breakage	3 (2.7%)
	Total	19 (16.8%)

**Table 4:** Overview of censored cases, cumulative events and remaining cases from the study sample in 6-month intervals.

Time (Months)	*N* of Censored Cases	*N* of Cumulative Events of Failure	*N* of Remaining Cases
6	17	7	89
12	38	11	64
18	52	15	46
20	56	15	42
24	67	16	30
30	75	17	21
36	88	19	6
45	93	19	1

## Data Availability

Public access to the data underlying this study is restricted due to privacy and data protection regulations specified in §§37–37d of the Landeskrankenhausgesetz Mecklenburg-Vorpommern (LKHG M-V). Access may be granted by the corresponding author upon reasonable request, provided that approval by the institutional ethics committee of University Medicine Greifswald and the local data protection authority is granted.

## Abbreviations

This manuscript includes the following abbreviations:

ASA	American Society of Anaesthesiologists
DSSM(s)	Distal Shoe space maintainer(s)
FPM(s)	First permanent molar(s)
PEPT	Premature extraction of primary teeth
SSC(s)	stainless steel crown(s)
